# Ca^2+^ signaling in the myocardium by (redox) regulation of PKA/CaMKII

**DOI:** 10.3389/fphar.2015.00166

**Published:** 2015-08-10

**Authors:** Alex S. Johnston, Stephan E. Lehnart, Joseph R. Burgoyne

**Affiliations:** ^1^Heart Research Center Goettingen, Clinic of Cardiology and Pulmonology, University Medical Center GoettingenGoettingen, Germany; ^2^German Center for Cardiovascular Research (DZHK) site GöttingenBerlin, Germany; ^3^Cardiovascular Division, The British Heart Foundation Centre of Excellence, The Rayne Institute, King's College London, St. Thomas' HospitalLondon, UK

**Keywords:** CaMKII, PKA, redox signaling, Ca^2+^ signaling, cardiac contractility

## Abstract

Homeostatic cardiac function is maintained by a complex network of interdependent signaling pathways which become compromised during disease progression. Excitation-contraction-coupling, the translation of an electrical signal to a contractile response is critically dependent on a tightly controlled sequence of events culminating in a rise in intracellular Ca^2+^ and subsequent contraction of the myocardium. Dysregulation of this Ca^2+^ handling system as well as increases in the production of reactive oxygen species (ROS) are two major contributing factors to myocardial disease progression. ROS, generated by cellular oxidases and by-products of cellular metabolism, are highly reactive oxygen derivatives that function as key secondary messengers within the heart and contribute to normal homeostatic function. However, excessive production of ROS, as in disease, can directly interact with kinases critical for Ca^2+^ regulation. This post-translational oxidative modification therefore links changes in the redox status of the myocardium to phospho-regulated pathways essential for its function. This review aims to describe the oxidative regulation of the Ca^2+^/calmodulin-dependent kinase II (CaMKII) and cAMP-dependent protein kinase A (PKA), and the subsequent impact this has on Ca^2+^ handling within the myocardium. Elucidating the impact of alterations in intracellular ROS production on Ca^2+^ dynamics through oxidative modification of key ROS sensing kinases, may provide novel therapeutic targets for preventing myocardial disease progression.

## ROS-sources and function

The sequential reduction of molecular oxygen leads to formation of biological forms of ROS comprising of the superoxide anion (O^−^_2_), hydrogen peroxide (H_2_O_2_) and hydroxyl radical (HO^∙^). These forms of ROS are capable of altering cellular signaling by modifying susceptible proteins. Initially thought to contribute solely to cellular damage it has become clear that ROS signaling pathways are in fact a highly organized and compartmentalized network critical for homeostatic biological function (Wu et al., 2010; Datla et al., 2014). For example ROS produced by nicotinamide adenine dinucleotide phosphate-oxidase (NOX) enzymes modulate cardiac transcription factors, cell migration, vascular tone, and cardiac contraction (Aslan and Ozben, 2003; Clempus et al., 2007; Burgoyne et al., 2012). However, their excessive production, both transiently, and chronically, are now implicated in numerous cardiovascular pathologies such as inflammation, arrhythmias, diabetes, hypertension, atherosclerosis, reperfusion injury, fibrosis, and diastolic dysfunction (Higashi et al., 2002; Fortuño et al., 2004; Zweier and Talukder, 2006; Birukov, 2009; Frantz et al., 2009; Giacco and Brownlee, 2010; Prysyazhna et al., 2012; Wagner et al., 2013; Murdoch et al., 2014).

Mitochondria are a major source of intracellular ROS generation due to leakage of electrons from the electron transport chain that react with molecular oxygen to generate superoxide. Once generated, the superoxide is readily converted to more stable H_2_O_2_ by the enzyme superoxide dismutase (SOD). Importantly, as mitochondrial complexes are abundant in heme groups and iron sulfur clusters H_2_O_2_ can also be converted to highly reactive hydroxyl radicals (Carroll et al., 2006; Chen and Zweier, 2014). In addition to ROS generated by the electron transport chain enzymatic networks such as NOX, xanthine oxidase and uncoupled nitric oxide synthases contribute to endogenous ROS production (Brown and Griendling, 2015). Homeostatic balance of this system is maintained through both enzymatic (SOD, catalase, glutathione peroxidase) and non-enzymatic [vitamins, thioredoxin (TRX), flavonoids] scavenging systems. Under normal homeostatic conditions this system is tightly balanced such that moderate increases in ROS can act as secondary messengers by reversibly modifying protein function. However, during disease states when ROS are excessively produced or when exogenously applied to mimic excess production, the innate anti-oxidant scavenging system becomes overwhelmed. The impact of this imbalance on the cellular environment is a shift to a state of excessive ROS levels termed “oxidative stress.”

While *in vivo* animal models of cardiac pathology have highlighted redox-balance as a mediator of heart failure progression the translation of this to a clinical setting is becoming more apparent. Congestive heart failure progression, dilated, and ischemic cardiomyopathy, end stage heart failure, chronic heart failure, and ethanol induced cardiac abnormalities are all associated with a concurrent rise in free radical generation (Jaatinen et al., 1993; Landmesser, 2002; Maack et al., 2003; Giordano, 2005; Kunishige et al., 2007; Nakamura et al., 2015; Shah, 2015). Additionally, it is emerging that the enzymes and cellular sources driving increased ROS production may be divergent depending on the pathology involved such as systolic vs. diastolic dysfunction (Münzel et al., 2015). However, long term controlled clinical antioxidant trials principally, using the antioxidants vitamin E, C, and β-carotene, have failed to find any evidence of a beneficial effect. In fact in some cases antioxidant therapy was seen to associate with morbidity and probability of a cardiac event (Kris-Etherton et al., 2004). While several hypotheses have been offered to explain this disappointing result the principal argument has been that exogenous application of anti-oxidants fails to effectively target discreet molecular sources of ROS production and may in fact hinder pro-survival oxidant signaling pathways. Therefore, in order to develop targeted and effective anti-oxidant therapies it is necessary to understand the molecular redox signaling pathways involved.

## Oxidative modification of proteins

The ability of ROS to act as secondary messengers is attributed to their ability to oxidize susceptible cysteine thiols or sulfur on methionine residues of select proteins. The alteration of cysteine or methionine residues by oxidation generates a conjugated moiety with a different shape and charge characteristic, which can induce structure rearrangement to alter enzymatic activity. Protein oxidation is considered a physiological signaling modality as it can specifically and transiently regulate protein function. The selectivity of protein oxidation is provided by the limited reactivity of cysteine thiols. This is due to the majority of cysteines being buried within proteins and thus not accessible to an oxidant. In addition the pKa of an accessible cysteine thiol is an important determinant of its reactivity. For a cysteine thiol to be sensitive to oxidation it generally needs to be in a deprotonated “reactive” thiolate anion form (S^−^) and thus have a low pKa. The pKa of a cysteine thiol is determined by its local tertiary environment, which is lowered by close proximity to basic amino acids lysine, arginine, or histidine.

**Figure 1:**
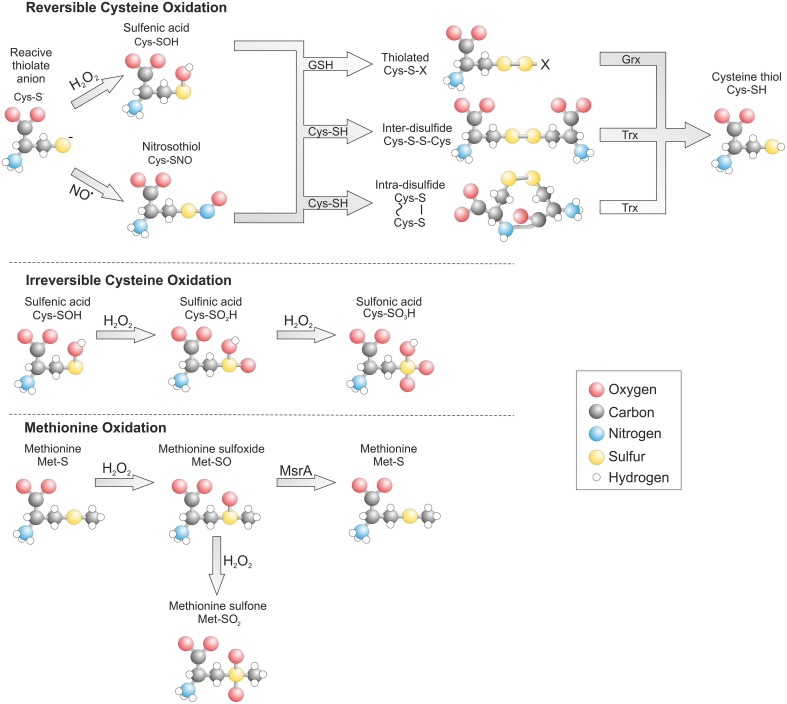
**Post translational oxidative modification of cysteine and methionine residues**. The gluthathiolation of a cysteine thiol can be reversibly reduced by glutaredoxin (Grx) while disulfides are reduced by thioredoxin (Trx). Oxidation of a methionine to the sulfoxide form can be reduced by methionine sulfoxide reductase A (MsrA).

A major form of ROS responsible for oxidant-dependent signaling is H_2_O_2_ formed enzymatically through the dismutation of superoxide. Once generated H_2_O_2_ readily reacts with cysteine thiols to form a sulfenic acid (SOH) intermediate, which is then rapidly resolved by an adjacent cysteine on the same protein or neighboring protein to form an intra- or inter-molecular disulfide respectively (Figure 1). Alternatively a sulfenic acid reacts with the highly replete cellular thiol containing tri-peptide glutathione (GSH), leading to protein glutathiolation.

Once oxidized a cysteine thiol can be reduced by the replete cellular reducing system. The rate of reduction and hence stability of an oxidative modification is determined by its type, accessibility and the quantity of oxidant present. Formation of an inter- or intra- molecular disulfide within a target protein can be reduced by TRX through a disulfide exchange reaction (Hanschmann et al., 2013). This leads to reduction of the target protein and oxidation of TRX, which is then recycled back to its reduced form by TRX reductase and electrons provided by NADPH. The formation of a glutathione adduct on a protein is also readily reversible and is enzymatically reduced by glutaredoxin. However, in contrast to TRX, glutaredoxin does not have an oxidoreductase and instead uses free GSH to reduce its target, which leads to formation of oxidized glutathione (GSSG). A similar mechanism for oxidation and reduction exists for methionine residues. When H_2_O_2_ reacts with methionine this generates a methionine sulfoxide than can be reduced by the aptly named methionine sulfoxide reductase (Msr) (Kim, 2013).

Under conditions of excessive oxidant production cysteine and methionine can transition to irreversibly oxidized states associated with disease and known as hyperoxidation. A cysteine sulfenic acid can be further oxidized to a sulfinic (SO_2_H) and then to sulfonic acid (SO_3_H). The formation of a sulfonic acid is irreversible, whereas a sulfinic acid is only reversible on peroxiredoxin at the expense of ATP and driven by the enzyme sulfiredoxin (Biteau et al., 2003). The reversibility of methionine sulfoxide is lost is the presence of excess H_2_O_2_, which leads to irreversible methionine sulfone formation.

In addition to H_2_O_2_ reactive nitrogen species (RNS) also contribute to thiol-dependent redox signaling by forming protein nitric oxide (NO) adducts termed S-nitrosylation (SNO). NO generated from L-arginine by the nitric oxide synthases while remaining in its native form is unable to directly modify cysteine thiols. However, the formation of the nitrosonium cation (NO^+^) or dinitrogen trioxide (N_2_O_3_) can induce direct thiol oxidation (N_2_O_3_ + RSH → RSNO + HNO_2_), whereas, small thiol containing compounds such as S-nitrosoglutathione (GSNO) can induce protein S-nitrosylation through an exchange reaction termed trans-nitrosylation (GSNO + RSH → RSNO + GSH) (Broniowska and Hogg, 2012). A protein nitrosothiol is relatively unstable and can therefore act as an intermediate in the formation of a more stable disulfide in a similar manner to a sulfenic acid. In situations where there is localized formation of both nitric oxide and superoxide this can lead to formation of the highly reactive species peroxynitrite (ONOO^−^). Peroxynitrite can induce S-nitrosylation but also irreversible tyrosine nitration that is often associated with disease.

The oxidative modifications induced by ROS and RNS account for a large proportion of cellular thiol-dependent redox signaling. However, this form of signaling is further extended by the ability of cysteines to be modified by reactive lipids and natural electrophiles. These modifications are predominantly dependent on the presence of an alpha-beta unsaturated carbonyl group that allows nucleophilic addition of a thiol through a Michael addition reaction. Examples of thiol reactive lipids include 15-deoxy-Δ12,14-Prostaglandin J2, nitro-oleic acid and 4-hydroxynonenal (Benderdour et al., 2003; Charles et al., 2011, 2014). Natural electrophiles that modify cysteines include the plant-derived compounds sulforaphane and curcumin (Dinkova-Kostova et al., 2002; Fang et al., 2005). Each of these thiol-reactive compounds has a biological effect on signaling dependent on their ability to modify cysteine thiols. However, even though they may share similar thiol-reactivity their target selectivity differs due to each compounds unique charge and shape characteristic, thus explaining why each has a varying physiological effect. As well as natural compounds a number of kinase inhibitors have been developed that target select cysteine thiols. This includes the ribosomal s6 kinase (RSK) inhibitor FMK and the transforming growth factor beta-activated kinase 1 inhibitor (5Z)-7-oxozeaenol (Cohen et al., 2005; Sogabe et al., 2015). The use of pharmacological inhibitors that modify a select thiol to inhibit its respective target provides further credence for protein oxidation being a selective mode of signaling.

## Methods for detecting protein oxidation

**Figure 2:**
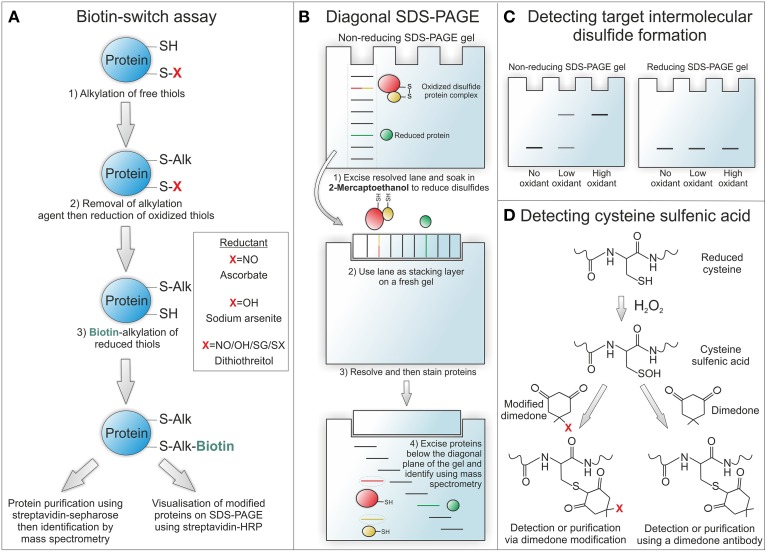
**Methods for detecting protein oxidation**. **(A)** The biotin-switch assay allows reversible oxidative modifications to be swapped for a detectable biotin-tag. To selectively detect S-nitrosothiol modified proteins ascorbate is used as a reducant and for sulfenic acids sodium arsenite. To detect all reversibly oxidized proteins dithiothreitol can be used as a reductant. **(B)** Diagonal SDS-PAGE is used to identify proteins that form intermolecular disulfide bound complexes. In the first dimension proteins are run under non-reducing conditions to maintain complexes at their combined disulfide-bound molecular weight. Once resolved the lane is excised and run on a second gel under reducing conditions so that disulfide bound proteins can then run at their individual unbound molecular weight. Once proteins have been resolved and then stained, those that were originally in a disulfide bound complex can be observed below the diagonal plane of the gel. These proteins can be identified by excising and then analysing by mass spectrometry. **(C)** Proteins already known to form intermolecular disulfide bound complexes or identified by diagonal SDS-PAGE can be analyzed in cell or tissue samples for covalent complex formation by resolving under non-reducing conditions. By omitting the reducing agent disulfide bound complexes are preserved and can be detected as a higher molecular weight complex on immunostained blots. The formation of higher molecular weight complexes will increase under oxidizing conditions due to enhanced disulfide formation and will be reducible with 2-mecaptoethanol. **(D)** Cysteine sulfenic acids can be detected by the addition of dimedone, as it is able to selectively bind to this modification. Fluorescent or biotin derivates of dimedone can be used for detection or purification of sulfenic acid modified proteins. Alternatively a dimedone specific antibody can be used for detecting this compound when bound to protein sulfenic acids.

The identification and characterization of protein oxidation has been largely hampered by a lack of direct methods for detection. Recent developments in novel tools and indirect methods for detecting protein oxidation have greatly improved the expanding field of redox biology (Figure 2). Many of these methods rely on exchange reactions, where the oxidative moiety is reduced and then swapped for a detectable tag that is usually biotin or in some cases a fluorophore (Burgoyne and Eaton, 2011). In this type of method, often referred to as biotin-switch, proteins (from lysate or homogenate) are firstly alkylated (usually with N-ethylmaleimide or maleimide) under denaturing conditions to block any free cysteine thiols. The alkylating reagent is then removed (usually by desalting), the oxidative moiety of interest selectively reduced and then these free thiols labeled with a biotin-tagged alkylating agent that is either modified maleimide, iodoacetamide, or pyridyldithiol. To examine S-nitrosylation ascorbate is used to selectively reduce this modification for labeling. For sulfenic acid formation sodium arsenite is used as a selective reductant and for total reversibly oxidized thiols either dithiothreitol, 2-mercaptoethanol or Tris(2-carboxyethyl)phosphine hydrochloride. Once the oxidative modification of interest has been exchanged for a biotin-tag, modified proteins can then be readily detected using streptavidin-HRP or purified using streptavidin sepharose and identified using mass spectrometry (Figure 2A). A recent variation on the biotin-switch assay is labeling oxidized thiols with maleimide conjugated to a 5 kDa PEG-tag (Burgoyne et al., 2013). This allows simple and rapid determination of target oxidation without the need for affinity purification. If a protein is oxidized it will be labeled following reduction with PEG-maleimide causing it to resolve at a higher molecular on a sodium dodecyl sulfate polyacrylamide gel electrophoresis (SDS-PAGE) gel. A change in the molecular weight of a target protein can then be determined by immunoblotting using a selective antibody. In addition to switch-based methods a number of novel dimedone-based tools have been developed to detect thiol sulfenic acids (Figure 2D). These methods exploit the specific reactivity of dimedone for sulfenic acids by using fluorescent and biotin derivates or antibodies for detection (Charles et al., 2007; Maller et al., 2011).

To identify novel proteins that form intermolecular disulfide complexes a method has been developed termed diagonal gel electrophoresis (Figure 2B) (Burgoyne and Eaton, 2013b). This method exploits the differential ability of proteins to resolve on an SDS-PAGE gel in their reduced and disulfide bound form. Firstly, samples are run under non-reducing conditions on a standard SDS-PAGE gel. Once fully resolved the lane is excised, soaked in 2-mercaptoethanol to reduce disulfides and then placed horizontally onto a new SDS-PAGE gel. This second gel is resolved and then proteins stained so that they can be visualized. The concept of this method is that disulfide bound complexes will resolve at a combined weight on the first gel and then reduced components at a lower molecular weight on the second gel. Therefore, any proteins originally in a disulfide bound complex will run off the diagonal plane of the second resolved gel. Once visualized any proteins running below the diagonal plane can be excised and indentified using mass spectrometry. This method was used to identify the regulatory R1 subunit of PKA, described below, as a redox-sensitive disulfide dimer (Brennan et al., 2006). PKARI was identified in an experiment using diamide to selectively induce disulfide formation in rat ventricular myocyte proteins before being resolved by diagonal gel electrophoresis.

Once a target of inter-molecular disulfide complex formation has been identified it can be readily screened in biological samples using a selective antibody. In this case tissue or cells should be lysed or homogenized into alkylating agent to prevent artificial air oxidation (Burgoyne and Eaton, 2013a). These samples are then run under non-reducing conditions and western blotted for the target of interest. If the target protein forms a disulfide bound complex then it will be detected at a higher molecular weight, which will be susceptible to reduction by 2-mercaptoethanol (Figure 2C). Examples of proteins that are known to form redox-regulated disulfide bound complexes in addition to PKARI include cyclic guanosine monophosphate (cGMP) dependent protein kinase 1α (PKG1α), mitsugumin 53, and peroxiredoxin (Burgoyne et al., 2007; Cai et al., 2009; Rhee and Woo, 2011).

## Excitation contraction coupling

Cardiac contraction is modulated through the coupling of an electrical stimulus to a contractile output in a process known as excitation-contraction coupling (ECC). An action potential depolarizes the cell membrane activating voltage sensitive L-type Ca^2+^ channels (LTCC/Cav1.2) and allowing Ca^2+^ to flow into the cell where it binds to cardiac ryanodine receptors (RyR2) on the adjacent sarcoplasmic reticulum (SR). This binding precipitates cell-wide Ca^2+^ release from the sarcoplasmic reticulum (SR) store termed “Ca^2+^ induced Ca^2+^ release” (CICR), giving rise to a synchronous cardiac contraction. For the myocardium to relax it is necessary that cytoplasmic Ca^2+^ levels are returned to diastolic levels allowing Ca^2+^ to dissociate from the myofilaments. This is accomplished through inactivation of extracellular Ca^2+^ entering through LTCC and Ca^2+^ extrusion from the cytoplasm via the sarcoplasmic reticulum ATPase (SERCA), Na/Ca^2+^ exchanger, sarcolemmal Ca^2+^ ATPase and mitochondrial uniporter (Bers, 2002).

A central tenet to myocardial dysfunction, heart failure progression and eventual cardiac arrest is the dysregulation of Ca^2+^ handling and thus ECC within cardiomyocytes. Following the development of cardiac or vascular pathology the contractile requirements of the heart are severely increased in order to adequately compensate for the diseased tissue. The abundance of LTCC is reduced and the channel becomes hyperphosphorylated (Chen, 2002; Lugenbiel et al., 2015), RyR2 are similarly hyperphosphorylated (Ai et al., 2005; Shan et al., 2010a), SERCA expression is reduced (Hasenfuss et al., 1994) as is its activity by hypo-phosphorylation of phospholamban (PLB) (Reiken et al., 2003) while NCX extrusion of Ca^2+^ is increased (Lugenbiel et al., 2015). The net impact of this, as seen in myocytes from failing hearts, is a reduced ability to drive Ca^2+^ into the cytoplasm and SR, therefore limiting Ca^2+^ release during systole and increasing its accumulation during diastole. Reduced systolic Ca^2+^ levels impair cardiac inotropy while its diastolic accumulation, through leak and inadequate re-uptake, is arrhythmogenic due to its extrusion via the electrogenic NCX which introduces a depolarizing current to the myocyte during diastole (Bers, 2014). Therefore, the failing myocardium must increase its contractile output in spite of a reduction in the basic machinery necessary to achieve this, and so the shift toward decompensated heart failure begins. It has become clear that aberrant oxidative-modification of proteins by excessive ROS production also contributes to dysfunctional Ca^2+^ handling and contractility in heart failure. As such any therapy aimed at correcting contractile abnormalities must also address specific redox signaling events.

Heart failure is associated with a shift in the redox environment of the heart to a pro-oxidative state. Although, there is evidence for a reduction in the antioxidant capacity of the heart during disease, the principal driving force toward this pro-oxidative milieu appears to be excessive ROS production (Landmesser, 2002; Maack et al., 2003; Giordano, 2005; Kunishige et al., 2007; Nakamura et al., 2015). To investigate the physiological consequence of this on the contractile ability of the myocardium, cardiomyocytes have been exposed to various forms of ROS, had oxidant formation inhibited or applied with exogenous anti-oxidants. Exposure of cardiomyocytes to H_2_O_2_, O^−^_2_ and HO^•^ decrease Ca^2+^ transient amplitude, SR Ca^2+^ content and SR Ca^2+^ re-uptake, indices that mimic that observed in the failing heart (Gill et al., 1995; Xu et al., 1998; Fearon et al., 1999). Additionally, counterbalancing ROS production through targeting of its sources or the application of anti-oxidants reverses contractile dysfunction (Shiomi et al., 2004; Guo et al., 2006; Gonzalez et al., 2007; Qin et al., 2007; Saliaris et al., 2007). These studies provide clear evidence that ROS directly impact the contractile ability of the myocardium. Furthermore, it has become apparent that the majority of channels involved in Ca^2+^ cycling are subject to oxidative modification.

The LTCC is the principal entry point of Ca^2+^ to the cytoplasm therefore any alterations in its flux will directly impact contraction. The α1c pore forming sub-unit of the LTCC is subject to direct oxidation by redox agents causing a decrease in peak Ca^2+^ entry (Gill et al., 1995; Fearon et al., 1999). The large tetrameric SR Ca^2+^ release protein RyR2 contains 89 cysteines per subunit, approximately 21 of which can be oxidized (Xu et al., 1998). Indeed, RyR2 immunoprecipitated from failing human myocardium show increased levels of oxidation and S-nitrosylation (Shan et al., 2010a). A graded response of RyR2 activity, Ca^2+^ release, in response to ROS exposure is reported and suggested as a fine-tuning mechanism under physiological conditions which becomes aberrant during excessive ROS exposure (Köhler et al., 2014). Importantly, diastolic leak of Ca^2+^ is known to contribute to the formation of Ca^2+^ waves, a precursor to the formation of lethal arrhythmic delayed-after-depolarizations. In addition leak via RyR2 will reduce SR Ca^2+^ content therefore attenuating cardiac inotropy. SERCA is responsible for shuttling Ca^2+^ out of the cytoplasm and into the SR. As such it is pivotal for determining the rate at which the myocardium can relax and the amount of Ca^2+^ available for subsequent contraction. Oxidation of SERCA at cysteine 674 is well described (Scherer and Deamer, 1986; Lancel et al., 2010). Treatment with oxidants or *in vivo* HF models inducing ROS production decrease its activity slowing Ca^2+^ sequestration into the SR (Qin et al., 2013, 2014). This reduces SR Ca^2+^ content and slows relaxation, both of which are characteristic trademarks of failing myocytes.

The activity of the NCX is observed to increase in HF (Pogwizd et al., 1999). Theoretically, enhanced reverse mode activity (Ca^2+^ influx to the cytoplasm) would be beneficial to the failing myocardium as it would compensate for low levels of systolic Ca^2+^. However, as the NCX also extrudes Ca^2+^ in exchange for Na^+^ its increased activity will introduce a depolarizing current to the cytosol, a mechanism contributing to the formation of arrhythmic delayed-after-depolarizations (Despa and Bers, 2013). Various ROS increase NCX activity (Reeves et al., 1986; Goldhaber, 1996; Kuster et al., 2010) therefore in HF this may assist low systolic Ca^2+^ levels but could also increase the propensity for arrhythmias. As with Ca^2+^, a number of channels tightly regulate Na^+^ balance within the myocardium. Through both ions' interaction with the NCX their fluctuations are tightly coupled to one another. Na_v_1.5 contains multiple methionine residues which upon oxidation dramatically slow its inactivation, prolonging Na^+^ influx (Kassmann et al., 2008). The key determinant of intracellular Na^+^ is the Na^+^/K^+^-ATPase which itself is also subject to inactivation by gluthathionylation of cysteine 46 (Figtree et al., 2009; Rasmussen et al., 2010). With ROS impacting numerous Ca^2+^ and Na^+^ handling channels critical for cardiac function it is clear that in heart failure their excess production will have profound impacts on the hearts contractile and electrical properties. Two kinases that can modulate the above channels and are themselves susceptible to activation by ROS are CaMKII and PKA, which are discussed in the following sections.

## Redox regulation of CamKII within the myocardium

CaMKII is a ubiquitously expressed multimeric serine/threonine kinase responsible for the phosphorylation of key ECC proteins as well as regulating gene transcription associated with pathological hypertrophy (Maier et al., 2003; Ai et al., 2005; Wagner et al., 2006; Zhang et al., 2015). Transgenic mice over expressing or lacking the predominant cardiac isoform (δc) of CaMKII as well as select single point mutations to its phosphorylation sites have implicated this kinase in diastolic dysfunction, hypertrophy, and arrhythmogenesis (Maier et al., 2003; Backs and Olson, 2006; Sossalla et al., 2011). These mechanisms are principally driven through its interaction with the critical Ca^2+^ handling proteins LTCC, RyR2, and PLB, the net effect of which is the excess diastolic accumulation of arrhythmogenic Ca^2+^ and Na^+^. CaMKII phosphorylation of PLB mediates Ca^2+^ sequestration into the SR by relieving its inhibitory effect on SERCA activity, thus potentially mitigating the accumulation of excessive diastolic Ca^2+^ levels.

Each monomer of the dodecameric CaMKII multimer associates via its C-terminal domain to form a double ringed hexameric complex (Rellos et al., 2010). The catalytic N-terminal of the kinase is responsible for substrate phosphorylation and is connected to the C-terminal via the regulatory domain (Rosenberg et al., 2005). Under basal conditions the regulatory subunit of each monomer acts as substrate for its catalytic counterpart. Additionally, the regulatory subunits flanking each catalytic subunit block the binding of ATP or substrate. Thus, under resting conditions the regulatory and catalytic domains remain closely associated in an auto-inhibited state. Essential to the activation of CaMKII is the binding of Ca^2+^ bound calmodulin (Ca-CaM) to the regulatory domain, which occurs under high intracellular Ca^2+^ concentrations. This induces a conformational shift in the structure of the kinase which disrupts the association between the regulatory and catalytic domains allowing the catalytic subunit to phosphorylate its substrates.

Activation of CaMKII by Ca-CaM is dependent on local Ca^2+^ concentration as well as the frequency of Ca^2+^ release events (De Koninck, 1998). High frequency oscillations in intracellular Ca^2+^ concentration, as occurs during tachycardia, lead to inter-subunit auto-phosphorylation at T287 (Erickson et al., 2011). The impact of this is a more than 1000-fold increase in the affinity of CaM for CaMKII (Meyer et al., 1992). Additionally, the presence of phosphate prevents the re-association of the catalytic and regulatory subunits thus maintaining the kinase in its active conformation (Lai et al., 1987). Importantly, this auto-activity is maintained even as Ca^2+^ levels return to basal conditions.

**Figure 3:**
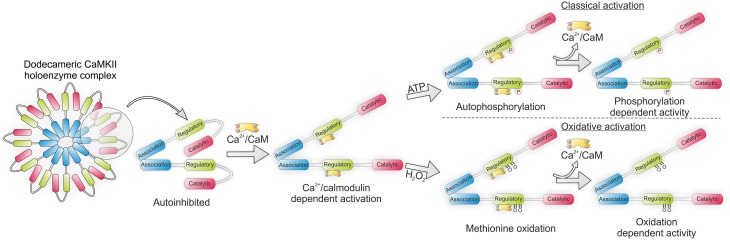
**CaMKII holoenzyme structure showing classical activation by autophosphorylation and oxidative activation by methionine sulfoxination**. In each instance binding of Ca^2+^/calmodulin (Ca-CaM) is necessary to first expose the regulatory domain.

Initial reports showing CaMKII to be active under pro-oxidant conditions such as during apoptosis led to the hypothesis that the kinase may be subject to oxidative modification. Indeed, CaMKII was found to remain active in myocytes in the presence of H_2_O_2_ and angiotensin II (AngII) even after the addition of a Ca^2+^ chelator (Palomeque et al., 2009). Crystal structure analysis of the kinase suggested that modification of methionine residues M281 and M282, located on the regulatory subunit of the kinase (Figure 3), may maintain the kinase in its active conformation, similarly to T287 auto-phosphorylation (Rellos et al., 2010). In support of this, an antibody generated to recognize dual sulfoxide modified Met281/282 showed that mutation of Met282Val completely inhibited the kinase's activation by oxidation while Met281Val partially attenuated this activation. In both cases pre-activation of the kinase with Ca^2+^/calmodulin was necessary to reveal the Met sites for modification. The application of AngII both *in vitro* and *in vivo* increased M281/282 oxidation resulting in apoptosis which was attenuated in M281/282V mice (Erickson et al., 2008). Similarly the cardiotoxic effects of aldosterone also appear to be mediated via CaMKII oxidation. Chronic treatment of mice with aldosterone following a myocardial infarction led to increased CaMKII oxidation and subsequent promotion of metalloproteinase 9, which was associated with increased mortality and cardiac rupture (He et al., 2011). Transgenic mice lacking MsrA are hyper-sensitized to CaMKII oxidation, myocyte apoptosis and structural remodeling, following a myocardial infarction while those over expressing it are protected from aldosterone mediated cardiac rupture (Erickson et al., 2008; He et al., 2011). The ability of oxidized CaMKII to interact with LTCC and modulate Ca^2+^ influx may account for some of its effects on the myocardium (Song et al., 2010).

Inflammation, an endogenous response triggered by tissue damage, that occurs after a myocardial infarction and ischemia reperfusion injury is associated with increased oxidative stress and ROS production (Frantz et al., 2009). In an *in vitro* model of myocyte inflammation using exposure to lipopolysaccharides CaMKII oxidation and activity increased while inhibition of the kinase lead to a reduction in the expression of pro-inflammatory genes (Singh et al., 2009). This relationship was then explored *in vivo* using myeloid differention protein 88 (MyD88) deficient mice. MyD88^−∕−^ myocytes showed no increase in CaMKII oxidation in response to lipopolysaccharide and were protected against CaMKII oxidation, cardiac hypertrophy, inflammation, apoptosis, and mortality following myocardial infarction (Singh et al., 2012). This suggests MyD88 mediated CaMKII oxidation may be an important regulator of cardiac hypertrophy and inflammation.

Alteration in the balance and flux of ions critical for the electrical stability of the heart, such as Ca^2+^ and Na^+^, fluctuate during pathology thus impacting on the susceptibility to fatal cardiac arrhythmias. Application of H_2_O_2_ to cardiac myocytes increases oxidation of CaMKII, the incidence of pro-arrhythmic Ca^2+^ sparks and late Na^+^ current leading to intracellular Na^+^ and Ca^2+^ overload (Wagner et al., 2011). Similarly, AngII was seen to modulate the activity of both PKA and CaMKII in a Nox2 dependent manner. This led to alterations in both Ca^2+^ and Na^+^ current which resulted in an increased propensity for cellular arrhythmias (Wagner et al., 2014). Using a Na^+^ channel enhancer to induce intracellular Na^+^ overload, this was observed to increase mitochondrial ROS production, CaMKII oxidation and subsequent Ca^2+^ dysregulation (Viatchenko-Karpinski et al., 2014). Indeed, levels of oxidized CaMKII are seen to be elevated in atrial fibrillation patients compared to those in sinus rhythm. In support of this mice infused with AngII have increased susceptibility to atrial fibrillation which is abolished in CaMKII oxidant-resistant (MM-VV) mice or those over-expressing cardiac specific MsrA (Purohit et al., 2013).

Levels of oxidized CaMKII are significantly increased in the pacemaker cells of diabetic patients following a myocardial infarction (Luo et al., 2013). This was also observed in pharmacologically induced diabetic mice receiving a myocardial infarction and was associated with pace maker cell apoptosis. Diabetic transgenic mice harboring CaMKII oxidant-resistant mutations MM-VV were protected against myocardial infarction, exhibiting reduced pace-maker cell apoptosis, heart rate, and disease associated mortality (Luo et al., 2013). Recently, CaMKII activation by O-linked N-acetlyglucosamine during acute hyperglycaemia was reported. This modification was observed in the hearts and brains of diabetic patients and rats and associated with increased arrhythmogenesis (Erickson et al., 2013). Therefore, a novel CaMKII- hyperglycaemia signaling pathway may have potential implications in cardiac and diabetic pathology.

A NO-dependent CaMKII activation pathway during β-adrenergic stimulation has been proposed. In this study β-adrenergic stimulation of cardiac myocytes increased NO production which activated CaMKII independently of Ca^2+^ transients leading to increased Ca^2+^ spark frequency. These findings were reproduced in the presence of GSNO and attenuated with specific CaMKII inhibitors. Additionally, S-nitrosylation of CaMKII was detectable using an antibody specific to S-nitrosated cysteines (Gutierrez et al., 2013). Recently, NO dependent S-nitrosylation of Cys280/289 has been shown to activate CaMKII. This was specific for the CaMKIIα isoform and dependent on S-nitrosylation of both Cys280 and 289 (Coultrap and Bayer, 2014). This is important to note as CaMKIIδ, the predominant cardiac isoform, lacks the homologous Cys280 of CaMKIIα. The authors therefore speculate that the observed increase in activation of cardiac CaMKII to NO donors in earlier reports (Gutierrez et al., 2013), may be independent of S-nitrosylation and instead due to other forms of oxidation mediated by ONOO^−^ production (Coultrap and Bayer, 2014). Therefore, whether cross-talk between β-adrenergic and NO signaling pathways have arrhythmogenic implications remains to be seen.

## Redox regulation of PKA within the myocardium

PKA is a heterotetrameric threonine/serine kinase composed of two regulatory and two catalytic subunits. The regulatory subunits of PKA exist in two isoforms RI and RII, the presence of either identifies its isozyme nomenclature as PKARI or PKARII. On binding of two cAMP molecules to the regulatory subunit the kinase undergoes a conformational change that releases its catalytic subunits allowing substrate phosphorylation. The regulatory subunits also contain an N-terminal dimerization/docking domain that dictates its localization via its interaction with A-kinase anchoring proteins (AKAPs), thus providing a mechanism for compartmentalization and specificity of PKA signaling (Kim et al., 2007).

Classically, PKA activation is modulated through the sympathetic nervous system. Upon binding of epinephrine or norepinephrine to G protein-coupled adrenergic receptors, adenylate cyclases are activated producing a rapid increase in intracellular cAMP. This rise in intracellular cAMP binds to the regulatory subunits of PKA activating the kinase (Benovic et al., 1988). The coupling of sympathetic innervation to intracellular PKA activity is responsible for increasing the hearts ability to adapt to higher energy demands such as in exercise and during pathology. PKA mediates this through its modulation of key ECC proteins to increase cardiac inotropy, lusitropy, and chronotropy (Bers, 2002).

Activated PKA may phosphorylate the LTCC to increase Ca^2+^ flux into the myoplasm (Hulme et al., 2003). However, the proposed LTCC phosphorylation sites are under debate (Hofmann et al., 2014). LTCC phosphorylation has been proposed to potentiate the release of Ca^2+^ from the SR through augmented CICR. Phosphorylation of troponin I by activated PKA increases the rate of Ca^2+^ dissociation from the myofilaments resulting in accelerated relaxation (lusitropy) (Layland et al., 2005). In addition to facilitating SR Ca^2+^ release PKA also augments Ca^2+^ sequestration into the SR by negatively regulating the inhibitory effect of PLB on SERCA (Li et al., 2000). This increased Ca^2+^ flux to the SR has the net effect of increasing SR Ca^2+^ content, SR Ca^2+^ release, and SR Ca^2+^ re-uptake thus PKA facilitates cardiac contraction, relaxation, and heart rate when activated (Bers, 2002). PKA has been shown to critically mediate RyR2 function via its specific S2808 phosphorylation site (Lehnart et al., 2006; Wehrens et al., 2006). Transgenic mice harboring a S2808A mutation show blunted inotropic and chronotropic response to catecholamines (Shan et al., 2010b), while phosphomimetic mutation of the receptor leads to age-dependent cardiomyopathy and arrhythmias (Shan et al., 2010a).

**Figure 4:**
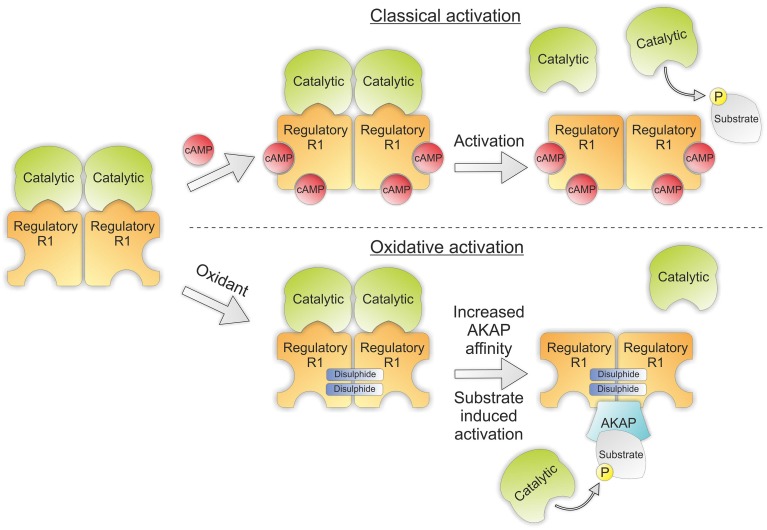
**PKA structure showing classical and oxidative activation**. Classical activation of PKA involves binding of cAMP to the regulatory subunits of the kinase which induces a conformational rearrangement releasing the catalytic subunits to phosphorylate their target substrates. Oxidative activation doesn't involve the binding of cAMP. Instead two intermolecular disulfides are formed between the regulatory subunits increasing their affinity for their corresponding AKAP. Interaction with their binding partners then brings PKA into proximity with its substrates, thus promoting substrate induced activation.

Importantly, the activation of PKA can be induced independently of cAMP by H_2_O_2_. This is through the formation of two inter-protein disulfide bonds between the RIα subunits (Figure 4) (Brennan et al., 2006). This bond was first identified following a proteomic screen and results in the formation of an anti-parallel dimer joined by two disulfides between Cys16 and Cys37 (Zick and Taylor, 1982). This bond was initially thought to be a constitutive modification due to the high concentration of reducing agent required to break it (Leon et al., 1997). In later studies the presence of disulfides within PKARI was evaluated by Langendorff perfusing isolated rat hearts with increasing concentrations of H_2_O_2_. These hearts were then homogenized in the presence of the alkylating agent maleimide to prevent artificial oxidation and PKARI resolved using SDS-PAGE in the absence of reducing agent. By using this approach a dose dependent increase in the percentage of disulfide bound RIα could be observed in response to H_2_O_2_ which was entirely abolished in the presence of reducing agent. Disulfide dimer formation was associated with translocation of the kinase to the myofilaments and phosphorylation of its targets troponin I and myosin binding protein C. This resulted in enhanced myocyte contractility independent of β-adrenergic stimulation or elevations in cAMP and was inhibited in the presence of the PKA inhibitor H89 (Brennan et al., 2006).

The translocation of disulfide bound PKARI to alternate subcellular locations may be explained by redox mediated changes in its affinity for AKAPs. Indeed, the cysteines which form disulfides in response to H_2_O_2_ directly flank the area of the regulatory subunit specifically responsible for AKAP binding. Evidence for this interaction is supported by studies utilizing mutant RIα with Cys17Ala or Cys37Ala which had 3 and 16 fold reductions respectively in their affinity for D-AKAP in comparison to wild-type RIα (Sarma et al., 2010). Additionally, it has been observed that the presence of PKA substrate itself can catalyze the separation of the regulatory and catalytic subunits of PKA (Vigil et al., 2005). This suggests a more intricate model of PKA-AKAP interaction may be warranted and may also explain how an increase in PKA substrate phosphorylation in response to peroxide can be observed despite no alterations in intracellular cAMP levels.

Additionally, nitro-cysteine (CysNO) can oxidize RIα resulting in vasodilation independent of cAMP or NO signaling pathways. Therefore, the activation of PKA by CysNO was anticipated to exert positive inotropic effects on the myocardium. However, this was not observed due to CysNO's concurrent oxidative activation of PKG1α which exerts a partial negative inotropic effect on the myocardium (Burgoyne and Eaton, 2009).

## Conclusion

The intracellular ROS environment is maintained through alterations in oxidant producing enzymes, peroxidases, and reducing proteins. Fluctuations in the redox status of biological systems directly and indirectly alter protein structure and function. Key mediators of cardiac physiological function that are subject to oxidative-modification are the kinases PKA and CaMKII. Oxidation of both kinases leads to their activation therefore translating intercellular oxidative fluctuations into phosphorylation-mediated signaling. Under conditions of excessive oxidant production these signaling pathways can become aberrant and may contribute to heart failure progression. Failure to thoroughly understand the precise chemical pathways underlying these changes may be responsible for the disappointing results of long-term anti-oxidant clinical trials. Full elucidation of the mechanisms involving protein oxidation will allow for the development of more specific therapies that may inhibit and/or cure cardiac disease progression.

### Conflict of interest statement

The authors declare that the research was conducted in the absence of any commercial or financial relationships that could be construed as a potential conflict of interest.

## References

[B1] Ai X. Curran J. W. Shannon T. R. Bers D. M. Pogwizd S. M. (2005). Ca2+/calmodulin-dependent protein kinase modulates cardiac ryanodine receptor phosphorylation and sarcoplasmic reticulum Ca2+ leak in heart failure. Circ. Res. 97, 1314–1322. 10.1161/01.RES.0000194329.41863.89 16269653

[B2] Aslan M. Ozben T. (2003). Oxidants in receptor tyrosine kinase signal transduction pathways. Antioxid. Redox Signal. 5, 781–788. 10.1089/152308603770380089 14588151

[B3] Backs J. Olson E. N. (2006). Control of cardiac growth by histone acetylation/deacetylation. Circ. Res. 98, 15–24. 10.1161/01.RES.0000197782.21444.8f 16397154

[B4] Benderdour M. Charron G. DeBlois D. Comte B. Des Rosiers C. (2003). Cardiac mitochondrial NADP+-isocitrate dehydrogenase is inactivated through 4-hydroxynonenal adduct formation: an event that precedes hypertrophy development. J. Biol. Chem. 278, 45154–45159. 10.1074/jbc.M306285200 12960146

[B5] Benovic J. L. Bouvier M. Caron M. G. Lefkowitz R. J. (1988). Regulation of adenylyl cyclase-coupled beta-adrenergic receptors. Annu. Rev. Cell Biol. 4, 405–428. 10.1146/annurev.cb.04.110188.002201 2848553

[B6] Bers D. M. (2002). Cardiac excitation-contraction coupling. Nature 415, 198–205. 10.1038/415198a 11805843

[B7] Bers D. M. (2014). Cardiac sarcoplasmic reticulum calcium leak: basis and roles in cardiac dysfunction. Annu. Rev. Physiol. 76, 107–127. 10.1146/annurev-physiol-020911-153308 24245942

[B8] Birukov K. G. (2009). Cyclic stretch, reactive oxygen species, and vascular remodeling. Antioxid. Redox Signal. 11, 1651–1667. 10.1089/ARS.2008.2390 19186986 PMC2842585

[B9] Biteau B. Labarre J. Toledano M. B. (2003). ATP-dependent reduction of cysteine-sulphinic acid by *S*. cerevisiae sulphiredoxin. Nature 425, 980–984. 10.1038/nature02075 14586471

[B10] Brennan J. P. Bardswell S. C. Burgoyne J. R. Fuller W. Schröder E. Wait R.. (2006). Oxidant-induced activation of type I protein kinase A is mediated by RI subunit interprotein disulfide bond formation. J. Biol. Chem. 281, 21827–21836. 10.1074/jbc.M603952200 16754666

[B11] Broniowska K. A. Hogg N. (2012). The chemical biology of S-nitrosothiols. Antioxid. Redox Signal. 17, 969–980. 10.1089/ars.2012.4590 22468855 PMC3411335

[B12] Brown D. I. Griendling K. K. (2015). Regulation of signal transduction by reactive oxygen species in the cardiovascular system. Circ. Res. 116, 531–549. 10.1161/CIRCRESAHA.116.303584 25634975 PMC4392388

[B13] Burgoyne J. R. Eaton P. (2009). Transnitrosylating nitric oxide species directly activate type I protein kinase A, providing a novel adenylate cyclase-independent cross-talk to beta-adrenergic-like signaling. J. Biol. Chem. 284, 29260–29268. 10.1074/jbc.M109.046722 19726669 PMC2785556

[B14] Burgoyne J. R. Eaton P. (2011). Contemporary techniques for detecting and identifying proteins susceptible to reversible thiol oxidation. Biochem. Soc. Trans. 39, 1260–1267. 10.1042/BST0391260 21936799

[B15] Burgoyne J. R. Eaton P. (2013a). Approaches for monitoring PKG1alpha oxidative activation. Methods Mol. Biol. 1020, 163–173. 10.1007/978-1-62703-459-3_10 23709032

[B16] Burgoyne J. R. Eaton P. (2013b). Detecting disulfide-bound complexes and the oxidative regulation of cyclic nucleotide-dependent protein kinases by H2O2. Meth. Enzymol. 528, 111–128. 10.1016/B978-0-12-405881-1.00007-0 23849862

[B17] Burgoyne J. R. Madhani M. Cuello F. Charles R. L. Brennan J. P. Schröder E.. (2007). Cysteine redox sensor in PKGIa enables oxidant-induced activation. Science 317, 1393–137. 10.1126/science.1144318 17717153

[B18] Burgoyne J. R. Oviosu O. Eaton P. (2013). The PEG-switch assay: a fast semi-quantitative method to determine protein reversible cysteine oxidation. J. Pharmacol. Toxicol. Methods 68, 297–301. 10.1016/j.vascn.2013.07.001 23856010

[B19] Burgoyne J. R. Prysyazhna O. Rudyk O. Eaton P. (2012). cGMP-dependent activation of protein kinase G precludes disulfide activation: implications for blood pressure control. Hypertension 60, 1301–1308. 10.1161/HYPERTENSIONAHA.112.198754 23006734

[B20] Cai C. Masumiya H. Weisleder N. Matsuda N. Nishi M. Hwang M.. (2009). MG53 nucleates assembly of cell membrane repair machinery. Nat. Cell Biol. 11, 56–64. 10.1038/ncb1812 19043407 PMC2990407

[B21] Carroll J. Fearnley I. M. Skehel J. M. Shannon R. J. Hirst J. Walker J. E. (2006). Bovine complex I is a complex of 45 different subunits. J. Biol. Chem. 281, 32724–32727. 10.1074/jbc.M607135200 16950771

[B22] Charles R. L. Burgoyne J. R. Mayr M. Weldon S. M. Hubner N. Dong H.. (2011). Redox regulation of soluble epoxide hydrolase by 15-deoxy-delta-prostaglandin J2 controls coronary hypoxic vasodilation. Circ. Res. 108, 324–334. 10.1161/CIRCRESAHA.110.235879 21164107 PMC3259859

[B23] Charles R. L. Rudyk O. Prysyazhna O. Kamynina A. Yang J. Morisseau C.. (2014). Protection from hypertension in mice by the Mediterranean diet is mediated by nitro fatty acid inhibition of soluble epoxide hydrolase. Proc. Natl. Acad. Sci. U.S.A. 111, 8167–8172. 10.1073/pnas.1402965111 24843165 PMC4050620

[B24] Charles R. L. Schröder E. May G. Free P. Gaffney P. R. Wait R.. (2007). Protein sulfenation as a redox sensor: proteomics studies using a novel biotinylated dimedone analogue. Mol. Cell. Proteomics 6, 1473–1484. 10.1074/mcp.M700065-MCP200 17569890

[B25] Chen X. (2002). L-Type Ca2+ channel density and regulation are altered in failing human ventricular myocytes and recover after support with mechanical assist devices. Circ. Res. 91, 517–524. 10.1161/01.res.0000033988.13062.7c 12242270

[B26] Chen Y. R. Zweier J. L. (2014). Cardiac mitochondria and reactive oxygen species generation. Circ. Res. 114, 524–537. 10.1161/CIRCRESAHA.114.300559 24481843 PMC4118662

[B27] Clempus R. E. Sorescu D. Dikalova A. E. Pounkova L. Jo P. Sorescu G. P.. (2007). Nox4 is required for maintenance of the differentiated vascular smooth muscle cell phenotype. Arterioscler Thromb. Vasc. Biol. 27, 42–48. 10.1161/01.ATV.0000251500.94478.18 17082491 PMC1868577

[B28] Cohen M. S. Zhang C. Shokat K. M. Taunton J. (2005). Structural bioinformatics-based design of selective, irreversible kinase inhibitors. Science 308, 1318–1321. 10.1126/science1108367 15919995 PMC3641834

[B29] Coultrap S. J. Bayer K. U. (2014). Nitric oxide induces Ca2+-independent activity of the Ca2+/calmodulin-dependent protein kinase II (CaMKII). J. Biol. Chem. 289, 19458–19465. 10.1074/jbc.M114.558254 24855644 PMC4094056

[B30] Datla S. R. McGrail D. J. Vukelic S. Huff L. P. Lyle A. N. Pounkova L.. (2014). Poldip2 controls vascular smooth muscle cell migration by regulating focal adhesion turnover and force polarization. Am. J. Physiol. Heart Circ. Physiol. 307, H945–H957. 10.1152/ajpheart.00918.2013 25063792 PMC4187069

[B31] De Koninck P. (1998). Sensitivity of CaM Kinase II to the frequency of Ca2+ oscillations. Science 279, 227–230. 10.1126/science.279.5348.227 9422695

[B32] Despa S. Bers D. M. (2013). Na(+) transport in the normal and failing heart - remember the balance. J. Mol. Cell. Cardiol. 61, 2–10. 10.1016/j.yjmcc.2013.04.011 23608603 PMC3720717

[B33] Dinkova-Kostova A. T. Holtzclaw W. D. Cole R. N. Itoh K. Wakabayashi N. Katoh Y.. (2002). Direct evidence that sulfhydryl groups of Keap1 are the sensors regulating induction of phase 2 enzymes that protect against carcinogens and oxidants. Proc. Natl. Acad. Sci. U.S.A. 99, 11908–11913. 10.1073/pnas.172398899 12193649 PMC129367

[B34] Erickson J. R. Joiner M. L. Guan X. Kutschke W. Yang J. Oddis C. V.. (2008). A dynamic pathway for calcium-independent activation of CaMKII by methionine oxidation. Cell 133, 462–474. 10.1016/j.cell.2008.02.048 18455987 PMC2435269

[B35] Erickson J. R. Patel R. Ferguson A. Bossuyt J. Bers D. M. (2011). Fluorescence resonance energy transfer-based sensor Camui provides new insight into mechanisms of calcium/calmodulin-dependent protein kinase II activation in intactcardiomyocytes. Circ.Res. 109, 729–738. 10.1161/circre-saha.111.247148 21835909 PMC3182829

[B36] Erickson J. R. Pereira L. Wang L. Han G. Ferguson A. Dao K.. (2013). Diabetic hyperglycaemia activates CaMKII and arrhythmias by O-linked glycosylation. Nature 502, 372–36. 10.1038/nature12537 24077098 PMC3801227

[B37] Fang J. Lu J. Holmgren A. (2005). Thioredoxin reductase is irreversibly modified by curcumin: a novel molecular mechanism for its anticancer activity. J. Biol. Chem. 280, 25284–25290. 10.1074/jbc.M414645200 15879598

[B38] Fearon I. M. Palmer A. C. V. Balmforth A. J. Ball S. G. Varadi G. Peers C. (1999). Modulation of recombinant human cardiac L-type Ca2+channel α1Csubunits by redox agents and hypoxia. J. Physiol. 514, 629–637. 10.1111/j.1469-7793.1999.629ad.x 9882735 PMC2269099

[B39] Figtree G. A. Liu C. C. Bibert S. Hamilton E. J. Garcia A. White C. N.. (2009). Reversible oxidative modification: a key mechanism of Na+-K+ pump regulation. Circ. Res. 105, 185–193. 10.1161/CIRCRESAHA.109.199547 19542013

[B40] Fortuño A. Oliván S. Beloqui O. San José G. Moreno M. U. Díez J.. (2004). Association of increased phagocytic NADPH oxidase-dependent superoxide production with diminished nitric oxide generation in essential hypertension. J. Hypertens. 22, 2169–2175. 10.1097/00004872-200411000-00020 15480102

[B41] Frantz S. Bauersachs J. Ertl G. (2009). Post-infarct remodelling: contribution of wound healing and inflammation. Cardiovasc. Res. 81, 474–481. 10.1093/cvr/cvn292 18977766 PMC2639128

[B42] Giacco F. Brownlee M. (2010). Oxidative stress and diabetic complications. Circ. Res. 107, 1058–1070. 10.1161/CIRCRESAHA.110.223545 21030723 PMC2996922

[B43] Gill J. S. McKenna W. J. Camm A. J. (1995). Free radicals irreversibly decrease Ca2+ currents in isolated guinea-pig ventricular myocytes. Eur. J. Pharmacol. 292, 337–340. 10.1016/0926-6917(95)90042-x 7796875

[B44] Giordano F. J. (2005). Oxygen, oxidative stress, hypoxia, and heart failure. J. Clin. Invest. 115, 500–508. 10.1172/JCI24408 15765131 PMC1052012

[B45] Goldhaber J. I. (1996). Free radicals enhance Na+/Ca2+ exchange in ventricular myocytes. Am. J. Physiol. 271 (3 Pt 2), H823–H833. 8853314 10.1152/ajpheart.1996.271.3.H823

[B46] Gonzalez D. R. Beigi F. Treuer A. V. Hare J. M. (2007). Deficient ryanodine receptor S-nitrosylation increases sarcoplasmic reticulum calcium leak and arrhythmogenesis in cardiomyocytes. Proc. Natl. Acad. Sci. U.S.A. 104, 20612–20617. 10.1073/pnas.0706796104 18077344 PMC2154479

[B47] Guo P. Nishiyama A. Rahman M. Nagai Y. Noma T. Namba T.. (2006). Contribution of reactive oxygen species to the pathogenesis of left ventricular failure in Dahl salt-sensitive hypertensive rats: effects of angiotensin II blockade. J. Hypertens. 24, 1097–1104. 10.1097/01.hjh.0000226200.73065.5d 16685210

[B48] Gutierrez D. A. Fernandez-Tenorio M. Ogrodnik J. Niggli E. (2013). NO-dependent CaMKII activation during beta-adrenergic stimulation of cardiac muscle. Cardiovasc. Res. 100, 392–401. 10.1093/cvr/cvt201 23963842

[B49] Hanschmann E. M. Godoy J. R. Berndt C. Hudemann C. Lillig C. H. (2013). Thioredoxins, glutaredoxins, and peroxiredoxins–molecular mechanisms and health significance: from cofactors to antioxidants to redox signaling. Antioxid. Redox Signal. 19, 1539–1605. 10.1089/ars.2012.4599 23397885 PMC3797455

[B50] Hasenfuss G. Reinecke H. Studer R. Meyer M. Pieske B. Holtz J.. (1994). Relation between myocardial function and expression of sarcoplasmic reticulum Ca(2+)-ATPase in failing and nonfailing human myocardium. Circ. Res. 75, 434–442. 10.1161/01.res.75.3.434 8062417

[B51] He B. J. Joiner M. L. Singh M. V. Luczak E. D. Swaminathan P. D. Koval O. M.. (2011). Oxidation of CaMKII determines the cardiotoxic effects of aldosterone. Nat. Med. 17, 1610–1618. 10.1038/nm.2506 22081025 PMC3332099

[B52] Higashi Y. Sasaki S. Nakagawa K. Matsuura H. Oshima T. Chayama K. (2002). Endothelial function and oxidative stress in renovascular hypertension. N. Engl. J. Med. 346, 1954–1962. 10.1056/NEJMoa013591 12075056

[B53] Hofmann F. Flockerzi V. Kahl S. Wegener J. W. (2014). L-type CaV1.2 calcium channels: from *in vitro* findings to *in vivo* function. Physiol. Rev. 94, 303–326. 10.1152/physrev.00016.2013 24382889

[B54] Hulme J. T. Lin T. W. Westenbroek R. E. Scheuer T. Catterall W. A. (2003). Beta-adrenergic regulation requires direct anchoring of PKA to cardiac CaV1.2 channels via a leucine zipper interaction with A kinase-anchoring protein 15. Proc. Natl. Acad. Sci. U.S.A. 100, 13093–13098. 10.1073/pnas.2135335100 14569017 PMC240750

[B55] Jaatinen P. Saukko P. Hervonen A. (1993). Chronic ethanol exposure increases lipopigment accumulation in human heart. Alcohol Alcohol. 28, 559–569. 8274180

[B56] Kassmann M. Hansel A. Leipold E. Birkenbeil J. Lu S. Q. Hoshi T.. (2008). Oxidation of multiple methionine residues impairs rapid sodium channel inactivation. Pflugers Arch. 456, 1085–1095. 10.1007/s00424-008-0477-6 18369661 PMC2913308

[B57] Kim C. Cheng C. Y. Saldanha S. A. Taylor S. S. (2007). PKA-I holoenzyme structure reveals a mechanism for cAMPdependent activation. Cell 130, 1032–1043. 10.1016/j.cell.2007.07.018 17889648

[B58] Kim H. Y. (2013). The methionine sulfoxide reduction system: selenium utilization and methionine sulfoxide reductase enzymes and their functions. Antioxid. Redox Signal. 19, 958–969. 10.1089/ars.2012.5081 23198996 PMC3763222

[B59] Köhler A. C. Sag C. M. Maier L. S. (2014). Reactive oxygen species and excitation-contraction coupling in the context of cardiac pathology. J. Mol. Cell. Cardiol. 73, 92–102. 10.1016/j.yjmcc.2014.03.001 24631768

[B60] Kris-Etherton P. M. Lichtenstein A. H. Howard B. V. Steinberg D. Witztum J. L. Nutrition Committee of the American Heart Association Council on Nutrition Physical Activity, Metabolism. (2004). Antioxidant vitamin supplements and cardiovascular disease. Circulation 110, 637–641. 10.1161/01.CIR.0000137822.39831.F1 15289389

[B61] Kunishige M. Kijima Y. Sakai T. Akutagawa O. Matsuo A. Nishibe A.. (2007). Transient enhancement of oxidant stress and collagen turnover in patients with acute worsening of congestive heart failure. Circ. J. 71, 1893–1897. 10.1253/circj.71.1893 18037742

[B62] Kuster G. M. Lancel S. Zhang J. Communal C. Trucillo M. P. Lim C. C.. (2010). Redox-mediated reciprocal regulation of SERCA and Na+-Ca2+ exchanger contributes to sarcoplasmic reticulum Ca2+ depletion in cardiac myocytes. Free Radic. Biol. Med. 48, 1182–1187. 10.1016/j.freeradbiomed.2010.01.038 20132882 PMC2847633

[B63] Lai Y. Nairn A. C. Gorelick F. Greengard P. (1987). Ca2+/calmodulin-dependent protein kinase II: identification of autophosphorylation sites responsible for generation of Ca2+/calmodulin-independence. Proc. Natl. Acad. Sci. U.S.A. 84, 5710–5714. 10.1073/pnas.84.16.5710 3475699 PMC298932

[B64] Lancel S. Qin F. Lennon S. L. Zhang J. Tong X. Mazzini M. J.. (2010). Oxidative posttranslational modifications mediate decreased SERCA activity and myocyte dysfunction in Galphaq-overexpressing mice. Circ. Res. 107, 228–232. 10.1161/CIRCRESAHA.110.217570 20508180 PMC2909347

[B65] Landmesser U. (2002). Vascular oxidative stress and endothelial dysfunction in patients with chronic heart failure: role of Xanthine-Oxidase and extracellular superoxide dismutase. Circulation 106, 3073–3078. 10.1161/01.cir.0000041431.57222.af 12473554

[B66] Layland J. Solaro R. J. Shah A. M. (2005). Regulation of cardiac contractile function by troponin I phosphorylation. Cardiovasc. Res. 66, 12–21. 10.1016/j.cardiores.2004.12.022 15769444

[B67] Lehnart S. E. Terrenoire C. Reiken S. Wehrens X. H. Song L. S. Tillman E. J.. (2006). Stabilization of cardiac ryanodine receptor prevents intracellular calcium leak and arrhythmias. Proc. Natl. Acad. Sci. U.S.A. 103, 7906–7910. 10.1073/pnas.0602133103 16672364 PMC1472543

[B68] León D. A. Herberg F. W. Banky P. Taylor S. S. (1997). A stable alpha-helical domain at the N terminus of the RIalpha subunits of cAMP-dependent protein kinase is a novel dimerization/docking motif. J. Biol. Chem. 272, 28431–28437. 9353302 10.1074/jbc.272.45.28431

[B69] Li L. Desantiago J. Chu G. Kranias E. G. Bers D. M. (2000). Phosphorylation of phospholamban and troponin I in beta-adrenergic-induced acceleration of cardiac relaxation. Am. J. Physiol. Heart Circ. Physiol. 278, H769–H779. Available online at: http://ajpheart.physiology.org/content/278/3/H769.long 10710345 10.1152/ajpheart.2000.278.3.H769

[B70] Lugenbiel P. Wenz F. Govorov K. Schweizer P. A. Katus H. A. Thomas D. (2015). Atrial fibrillation complicated by heart failure induces distinct remodeling of calcium cycling proteins. PLoS ONE 10:e0116395. 10.1371/journal.pone.0116395 25775120 PMC4361185

[B71] Luo M. Guan X. Luczak E. D. Lang D. Kutschke W. Gao Z.. (2013). Diabetes increases mortality after myocardial infarction by oxidizing CaMKII. J. Clin. Invest. 123, 1262–1274. 10.1172/JCI65268 23426181 PMC3673230

[B72] Maack C. Kartes T. Kilter H. Schäfers H. J. Nickenig G. Bohm M.. (2003). Oxygen free radical release in human failing myocardium is associated with increased activity of rac1-GTPase and represents a target for statin treatment. Circulation 108, 1567–1574. 10.1161/01.CIR.0000091084.46500.BB 12963641

[B73] Maier L. S. Zhang T. Chen L. DeSantiago J. Brown J. H. Bers D. M. (2003). Transgenic CaMKIIdeltaC overexpression uniquely alters cardiac myocyte Ca2+ handling: reduced SR Ca2+ load and activated SR Ca2+ release. Circ. Res. 92, 904–911. 10.1161/01.RES.0000069685.20258.F1 12676813

[B74] Maller C. Schröder E. Eaton P. (2011). Glyceraldehyde 3-phosphate dehydrogenase is unlikely to mediate hydrogen peroxide signaling: studies with a novel anti-dimedone sulfenic acid antibody. Antioxid. Redox Signal. 14, 49–60. 10.1089/ars.2010.3149 20518697

[B75] Meyer T. Hanson P. I. Stryer L. Schulman H. (1992). Calmodulin trapping by calcium-calmodulin-dependent protein kinase. Science 256, 1199–202. 10.1126/science.256.5060.1199 1317063

[B76] Münzel T. Gori T. Keaney J. F. Jr. Maack C. Daiber A. (2015). Pathophysiological role of oxidative stress in systolic and diastolic heart failure and its therapeutic implications. Eur. Heart J. [Epub ahead of print]. 10.1093/eurheartj/ehv305 26142467 PMC7959410

[B77] Murdoch C. E. Chaubey S. Zeng L. Yu B. Ivetic A. Walker S. J.. (2014). Endothelial NADPH oxidase-2 promotes interstitial cardiac fibrosis and diastolic dysfunction through proinflammatory effects and endothelial-mesenchymal transition. J. Am. Coll. Cardiol. 63, 2734–2741. 10.1016/j.jacc.2014.02.572 24681145

[B78] Nakamura T. Ranek M. J. Lee D. I. Shalkey Hahn V. Kim C. Eaton P.. (2015). Prevention of PKG1alpha oxidation augments cardioprotection in the stressed heart. J. Clin. Invest. 125, 2468–2472. 10.1172/JCI80275 25938783 PMC4497760

[B79] Palomeque J. Rueda O. V. Sapia L. Valverde C. A. Salas M. Petroff M. V.. (2009). Angiotensin II-induced oxidative stress resets the Ca2+ dependence of Ca2+-calmodulin protein kinase II and promotes a death pathway conserved across different species. Circ. Res. 105, 1204–1212. 10.1161/CIRCRESAHA.109.204172 19850941

[B80] Pogwizd S. M. Qi M. Yuan W. Samarel A. M. Bers D. M. (1999). Upregulation of Na+/Ca2+ exchanger expression and function in an arrhythmogenic rabbit model of heart failure. Circ. Res. 85, 1009–1019. 10.1161/01.res.85.11.1009 10571531

[B81] Prysyazhna O. Rudyk O. Eaton P. (2012). Single atom substitution in mouse protein kinase G eliminates oxidant sensing to cause hypertension. Nat. Med. 18, 286–290. 10.1038/nm.2603 22245782 PMC3276848

[B82] Purohit A. Rokita A. G. Guan X. Chen B. Koval O. M. Voigt N.. (2013). Oxidized Ca(2+)/calmodulin-dependent protein kinase II triggers atrial fibrillation. Circulation 128, 1748–1757. 10.1161/CIRCULATIONAHA.113.003313 24030498 PMC3876034

[B83] Qin F. Simeone M. Patel R. (2007). Inhibition of NADPH oxidase reduces myocardial oxidative stress and apoptosis and improves cardiac function in heart failure after myocardial infarction. Free Radic. Biol. Med. 43, 271–281. 10.1016/j.freeradbiomed.2007.04.021 17603936

[B84] Qin F. Siwik D. A. Lancel S. Zhang J. Kuster G. M. Luptak I.. (2013). Hydrogen peroxide-mediated SERCA cysteine 674 oxidation contributes to impaired cardiac myocyte relaxation in senescent mouse heart. J. Am. Heart Assoc. 2:e000184. 10.1161/JAHA.113.000184 23963753 PMC3828801

[B85] Qin F. Siwik D. A. Pimentel D. R. Morgan R. J. Biolo A. Tu V. H.. (2014). Cytosolic H2O2 mediates hypertrophy, apoptosis, and decreased SERCA activity in mice with chronic hemodynamic overload. Am. J. Physiol. Heart Circ. Physiol. 306, H1453–H1463. 10.1152/ajpheart.00084.2014 24633550 PMC4024717

[B86] Rasmussen H. H. Hamilton E. J. Liu C. C. Figtree G. A. (2010). Reversible oxidative modification: implications for cardiovascular physiology and pathophysiology. Trends Cardiovasc. Med. 20, 85–90. 10.1016/j.tcm.2010.06.002 21130951

[B87] Reeves J. P. Bailey C. A. Hale C. C. (1986). Redox modification of sodium-calcium exchange activity in cardiac sarcolemmal vesicle. J. Biol. Chem. 261, 4948–4955. 3007482

[B88] Reiken S. Gaburjakova M. Guatimosim S. Gomez A. M. D'Armiento J. Burkhoff D.. (2003). Protein kinase A phosphorylation of the cardiac calcium release channel (ryanodine receptor) in normal and failing hearts. Role of phosphatases and response to isoproterenol. J. Biol. Chem. 278, 444–453. 10.1074/jbc.M207028200 12401811

[B89] Rellos P. Pike A. C. Niesen F. H. Salah E. Lee W. H. von Delft F.. (2010). Structure of the CaMKIIdelta/calmodulin complex reveals the molecular mechanism of CaMKII kinase activation. PLoS Biol. 8:e1000426. 10.1371/journal.pbio.1000426 20668654 PMC2910593

[B90] Rhee S. G. Woo H. A. (2011). Multiple functions of peroxiredoxins: peroxidases, sensors and regulators of the intracellular messenger H(2)O(2), and protein chaperones. Antioxid. Redox Signal. 15, 781–794. 10.1089/ars.2010.3393 20919930

[B91] Rosenberg O. S. Deindl S. Sung R. J. Nairn A. C. Kuriyan J. (2005). Structure of the autoinhibited kinase domain of CaMKII and SAXS analysis of the holoenzyme. Cell 123, 849–860. 10.1016/j.cell.2005.10.029 16325579

[B92] Saliaris A. P. Amado L. C. Minhas K. M. Schuleri K. H. Lehrke S. St John M.. (2007). Chronic allopurinol administration ameliorates maladaptive alterations in Ca2+ cycling proteins and beta-adrenergic hyporesponsiveness in heart failure. Am. J. Physiol. Heart Circ. Physiol. 292, H1328–H1335. 10.1152/ajpheart.00461.2006 17071724

[B93] Sarma G. N. Kinderman F. S. Kim C. von Daake S. Chen L. Wang B. C.. (2010). Structure of D-AKAP2:PKA RI complex: insights into AKAP specificity and selectivity. Structure 18, 155–166. 10.1016/j.str.2009.12.012 20159461 PMC3090270

[B94] Scherer N. M. Deamer D. W. (1986). Oxidative stress impairs the function of sarcoplasmic reticulum by oxidation of sulfhydryl groups in the Ca2+-ATPase. Arch. Biochem. Biophys. 246, 589–601. 10.1016/0003-9861(86)90314-0 2939799

[B95] Shah A. M. (2015). Parsing the role of NADPH oxidase enzymes and reactive oxygen species in heart failure. Circulation 131, 602–64. 10.1161/CIRCULATIONAHA.115.014906 25589558

[B96] Shan J. Betzenhauser M. J. Kushnir A. Reiken S. Meli A. C. Wronska A.. (2010a). Role of chronic ryanodine receptor phosphorylation in heart failure and beta-adrenergic receptor blockade in mice. J. Clin. Invest. 120, 4375–4387. 10.1172/JCI37649 21099115 PMC2993577

[B97] Shan J. Kushnir A. Betzenhauser M. J. Reiken S. Li J. Lehnart S. E.. (2010b). Phosphorylation of the ryanodine receptor mediates the cardiac fight or flight response in mice. J. Clin. Invest. 120, 4388–4398. 10.1172/JCI32726 21099118 PMC2993575

[B98] Shiomi T. Tsutsui H. Matsusaka H. Murakami K. Hayashidani S. Ikeuchi M.. (2004). Overexpression of glutathione peroxidase prevents left ventricular remodeling and failure after myocardial infarction in mice. Circulation 109, 544–549. 10.1161/01.CIR.0000109701.77059.E9 14744974

[B99] Singh M. V. Kapoun A. Higgins L. Kutschke W. Thurman J. M. Zhang R. (2009). Ca2+/calmodulin-dependent kinase II triggers cell membrane injury by inducing complement factor B gene expression in the mouse heart. J. Clin. Invest. 119, 986–996. 10.1172/JCI35814 19273909 PMC2662543

[B100] Singh M. V. Swaminathan P. D. Luczak E. D. Kutschke W. Weiss R. M. Anderson M. E. (2012). MyD88 mediated inflammatory signaling leads to CaMKII oxidation, cardiac hypertrophy and death after myocardial infarction. J. Mol. Cell. Cardiol. 52, 1135–1144. 10.1016/j.yjmcc.2012.01.021 22326848 PMC3327770

[B101] Sogabe Y. Matsumoto T. Hashimoto T. Kirii Y. Sawa M. Kinoshita T. (2015). 5Z-7-Oxozeaenol covalently binds to MAP2K7 at Cys218 in an unprecedented manner. Bioorg. Med. Chem. Lett. 25, 593–596. 10.1016/j.bmcl.2014.12.011 25529738

[B102] Song Y. H. Cho H. Ryu S. Y. Yoon J. Y. Park S. H. Noh C. I.. (2010). L-type Ca(2+) channel facilitation mediated by H(2)O(2)-induced activation of CaMKII in rat ventricular myocytes. J. Mol. Cell. Cardiol. 48, 773–780. 10.1016/j.yjmcc.2009.10.020 19883656

[B103] Sossalla S. Maurer U. Schotola H. Hartmann N. Didie M. Zimmermann W. H. (2011). Diastolic dysfunction and arrhythmias caused by overexpression of CaMKIIdelta(C) can be reversed by inhibition of late Na+ current. Basic Res. Cardiol. 106, 263–272. 10.1007/s00395-010-0136-x 21174213 PMC3032905

[B104] Viatchenko-Karpinski S. Kornyeyev D. El-Bizri N. Budas G. Fan P. Jiang Z.. (2014). Intracellular Na+ overload causes oxidation of CaMKII and leads to Ca2+ mishandling in isolated ventricular myocytes. J. Mol. Cell. Cardiol. 76, 247–256. 10.1016/j.yjmcc.2014.09.009 25252177 PMC4250389

[B105] Vigil D. Blumenthal D. K. Taylor S. S. Trewhella J. (2005). The conformationally dynamic C helix of the RIalpha subunit of protein kinase A mediates isoform-specific domain reorganization upon C subunit binding. J. Biol. Chem. 280, 35521–35527. 10.1074/jbc.M506769200 16109722

[B106] Wagner S. Dantz C. Flebbe H. Azizian A. Sag C. M. Engels S.. (2014). NADPH oxidase 2 mediates angiotensin II-dependent cellular arrhythmias via PKA and CaMKII. J. Mol. Cell. Cardiol. 75, 206–215. 10.1016/j.yjmcc.2014.07.011 25073061

[B107] Wagner S. Dybkova N. Rasenack E. C. Jacobshagen C. Fabritz L. Kirchhof P.. (2006). Ca2+/calmodulin-dependent protein kinase II regulates cardiac Na+ channels. J. Clin. Invest. 116, 3127–3138. 10.1172/JCI26620 17124532 PMC1654201

[B108] Wagner S. Rokita A. G. Anderson M. E. Maier L. S. (2013). Redox regulation of sodium and calcium handling. Antioxid. Redox Signal. 18, 1063–1077. 10.1089/ars.2012.4818 22900788 PMC3567778

[B109] Wagner S. Ruff H. M. Weber S. L. Bellmann S. Sowa T. Schulte T. Anderson M. E.. (2011). Reactive oxygen species-activated Ca/calmodulin kinase IIδ is required for late I(Na) augmentation leading to cellular Na and Ca overload. Circ. Res. 108, 555–565. 10.1161/CIRCRESAHA.110.221911 21252154 PMC3065330

[B110] Wehrens X. H. Lehnart S. E. Reiken S. Vest J. A. Wronska A. Marks A. R. (2006). Ryanodine receptor/calcium release channel PKA phosphorylation: a critical mediator of heart failure progression. Proc. Natl. Acad. Sci. U.S.A. 103, 511–518. 10.1073/pnas.0510113103 16407108 PMC1334677

[B111] Wu R. F. Ma Z. Liu Z. Terada L. S. (2010). Nox4-derived H2O2 mediates endoplasmic reticulum signaling through local Ras activation. Mol. Cell Biol. 30, 3553–3568. 10.1128/MCB.01445-09 20457808 PMC2897542

[B112] Xu L. Eu J. P. Meissner G. Stamler J. S. (1998). Activation of the cardiac calcium release channel (ryanodine receptor) by poly-S-nitrosylation. Science 279, 234–237. 9422697 10.1126/science.279.5348.234

[B113] Zhang M. Hagenmueller M. Riffel J. H. Kreusser M. M. Bernhold E. Fan J.. (2015). Calcium/calmodulin-dependent protein kinase II couples Wnt signaling with histone deacetylase 4 and mediates dishevelled-induced cardiomyopathy. Hypertension 65, 335–344. 10.1161/HYPERTENSIONAHA.114.04467 25489064

[B114] Zick S. K. Taylor S. S. (1982). Interchain disulfide bonding in the regulatory subunit of cAMP-dependent protein kinase I. J. Biol. Chem. 257, 2287–2293 6277892

[B115] Zweier J. L. Talukder M. A. (2006). The role of oxidants and free radicals in reperfusion injury. Cardiovasc. Res. 70, 181–190. 10.1016/j.cardiores.2006.02.025 16580655

